# Molecular detection of thyroglobulin mRNA transcripts in peripheral blood of patients with thyroid disease by RT-PCR

**DOI:** 10.1054/bjoc.1999.1209

**Published:** 2000-04-27

**Authors:** J Bojunga, S Röddiger, M Stanisch, K Kusterer, R Kurek, H Renneberg, S Adams, E Lindhorst, K H Usadel, P M Schumm-Draeger

**Affiliations:** Department of Medicine I, JW Goethe-University, Theodor-Stern-Kai7, Frankfurt am Main, 60590; Department of Urology, Städtische Kliniken Offenbach, Offenbach am Main; Department of Anatomy and Cell Biology, Philipps-University, Marburg; Department of Nuclear Medicine and Department of Surgery, JW Goethe-University, Frankfurt am Main, Germany

**Keywords:** polymerase chain reaction, reverse transcription, thyroid cancer, thyroglobulin mRNA, molecular diagnostics

## Abstract

The sensitive detection of circulating tumour cells in patients with differentiated thyroid cancer may precede the detection of relapse by other diagnostic studies – such as serum thyroglobulin – and thus may have important therapeutic and prognostic implications. We performed reverse transcription-polymerase chain reaction (RT-PCR) on blood samples from patients diagnosed with thyroid disease using two different RT-PCR sensitivities. Additionally, tissue specificity of TG mRNA-expression was determined using RNA extracts from 27 different human tissues. The lower limit of detection was 50–100 TG mRNA producing cells/ml blood using a ‘normal’ RT-PCR sensitivity and 10–20 cells/ml blood using a ‘high’ sensitivity. With the normal sensitivity TG mRNA was detected in 9/13 patients with thyroid cancer and metastasis, 63/137 patients with a history of thyroid cancer and no metastasis, 21/85 with non-malignant thyroid disease and 9/50 controls. With the high sensitivity TG mRNA was detected in 11/13 patients with thyroid cancer and metastasis, 111/137 patients with a history of thyroid cancer and no metastasis, 61/85 with non-malignant thyroid disease and 41/50 controls. Interestingly, using the normal RT-PCR sensitivity TG mRNA transcripts are specific for thyroid tissue and detectable in the peripheral blood of controls and patients with thyroid disease, which correlates with a diagnosis of metastasized thyroid cancer. However, with a high RT-PCR sensitivity, TG mRNA expression was found not to be specific for thyroid tissue and was not correlated with a diagnosis of thyroid cancer in patients. As a consequence, to date TG mRNA detected by RT-PCR in the peripheral blood cannot be recommended as a tumour marker superior to TG serum-level. © 2000 Cancer Research Campaign


*British Journal of Cancer* (2000) **82**(10), 1650–1655
© 2000 Cancer Research Campaign
DOI: 10.1054/ bjoc.1999.1209, available online at http://www.idealibrary.com on IDEAL®

### Molecular detection of thyroglobulin mRNA transcripts in peripheral blood of patients with thyroid disease by RT-PCR

**J Bojunga[1], S Röddiger[1], M Stanisch[1], K Kusterer[1], R Kurek[2], H Renneberg[3], S Adams[4], E Lindhorst[5], KH Usadel[1] and PM Schumm-Draeger[1]**

[1] Department of Medicine I, JW Goethe-University, Theodor-Stern-Kai7, 60590 Frankfurt am Main; [2] Department of Urology, Städtische Kliniken Offenbach, Offenbach am Main; [3] Department of Anatomy and Cell Biology, Philipps-University, Marburg; [4] Department of Nuclear Medicine, and [5] Department of Surgery, JW Goethe-University, Frankfurt am Main; Germany

**Summary** The sensitive detection of circulating tumour cells in patients with differentiated thyroid cancer may precede the detection of relapse by other diagnostic studies – such as serum thyroglobulin – and thus may have important therapeutic and prognostic implications. We performed reverse transcription-polymerase chain reaction (RT-PCR) on blood samples from patients diagnosed with thyroid disease using two different RT-PCR sensitivities. Additionally, tissue specificity of TG mRNA-expression was determined using RNA extracts from 27 different human tissues. The lower limit of detection was 50–100 TG mRNA producing cells/ml blood using a 'normal' RT-PCR sensitivity and 10–20 cells/ml blood using a 'high' sensitivity. With the normal sensitivity TG mRNA was detected in 9/13 patients with thyroid cancer and metastasis, 63/137 patients with a history of thyroid cancer and no metastasis, 21/85 with non-malignant thyroid disease and 9/50 controls. With the high sensitivity TG mRNA was detected in 11/13 patients with thyroid cancer and metastasis, 111/137 patients with a history of thyroid cancer and no metastasis, 61/85 with non-malignant thyroid disease and 41/50 controls. Interestingly, using the normal RT-PCR sensitivity TG mRNA transcripts are specific for thyroid tissue and detectable in the peripheral blood of controls and patients with thyroid disease, which correlates with a diagnosis of metastasized thyroid cancer. However, with a high RT-PCR sensitivity, TG mRNA expression was found not to be specific for thyroid tissue and was not correlated with a diagnosis of thyroid cancer in patients. As a consequence, to date TG mRNA detected by RT-PCR in the peripheral blood cannot be recommended as a tumour marker superior to TG serum-level. © 2000 Cancer Research Campaign

**Keywords:** polymerase chain reaction; reverse transcription; thyroid cancer; thyroglobulin mRNA; molecular diagnostics

Differentiated thyroid cancer is the most common endocrine malignancy (Simpson et al, 1987) and being diagnosed with increasing frequency in the USA (Parker et al, 1997). Despite advances in the treatment of thyroid cancer, disease recurrence and metastasis may occur in as many as 20% of patients (Loh et al, 1997) and so continue to pose major problems in clinical management.

Before metastasis in a distant site can develop, tumour cells must circulate in the peripheral blood or lymphatic channels. Although the relationship between circulating tumour cells and the development of secondary disease is not fully understood (Johnson et al, 1995), a method to detect small numbers of such cells may provide a tool for the early diagnosis of tumour metastases to better define prognostic groups (Schlumberger et al, 1986; Sanders and Cady, 1998). This is of particular interest, because the presence of metastases is an important prognostic indicator (Simpson et al, 1987; Bellantone et al, 1998) and effective treatment modalities are available (Noguchi et al, 1998). Reverse transcription polymerase chain reaction (RT-PCR) is a highly sensitive technique to detect tissue-specific messenger ribonucleic acid (mRNA) expression. This approach has already been used to identify micro-metastases of different solid tumours such as prostate cancer (Ghossein et al, 1995) and neuroblastoma (Mattano et al, 1992). RT-PCR is also discussed as a new diagnostic tool with increasing popularity for the clinical monitoring of thyroid cancer patients (Haber, 1998). Controversial results were published by several groups during the last years and no standardized protocol is established to date (Ditkoff et al, 1996; Tallini et al, 1998; Ringel et al, 1998). To further define the applicability and value of this method we amplified thyroglobulin (TG) mRNA transcripts from peripheral blood samples of 150 patients diagnosed with thyroid cancer, 85 patients with non-malignant thyroid disease and 50 control subjects without evidence or history of thyroid disease. All experiments were performed using two different RT-PCR sensitivities. We report here that the detection of TG mRNA-producing cells in the peripheral blood and the correlation with extrathyroidal disease is crucially dependent on the used RT-PCR assay sensitivity. In addition, we report that tissue specific detection of thyroglobulin mRNA expression in different human tissues is also dependent on assay sensitivity. However, using our normal assay sensitivity it is possible to correlate the RT-PCR results with the diagnosis of metastasized thyroid cancer.

*Received 7 July 1999*
*Revised 16 November 1999*
*Accepted 20 December 1999*

*Correspondence to:* J Bojunga

### METHODS

### Patients

After approval by the human study committee, 150 patients with thyroid cancer (137 without current metastasis, 13 with metastasis), 29 patients with goitre, 56 patients with autoimmune disease of the thyroid (Graves' disease, Hashimoto thyroiditis) and

**Table 1** Oligonucleotides and primers

| Oligonucleotide | Sequence | Size of amplicon | Gene Bank accession |
|---|---|---|---|
| Thyroglobulin primer (+) | 5′CCTTGTTTGTCCCTGCTTGT 3′ | 219 | U93033 |
| Thyroglobulin primer (–) | 5′GTGGGTAGCATGCTGGAGTT 3′ | | |
| GAPDH primer (+) | 5′CGTCTTCACCACCATGGAGA 3′ | 300 | M33197 |
| GAPDH primer (–) | 5′CGGCCATCACGCCACAGTTT 3′ | | |

50 control subjects without evidence or history of thyroid disease, were studied. Diagnosis of cancer was based on histological examination of the surgical specimen, diagnosis of autoimmune disease was based on ultrasound examination and the presence of corresponding antibodies. Diagnosis of goitre as well as of healthy individuals was based on an appropriate ultrasound examination and normal serum markers (thyroid-stimulating hormone (TSH), total thyroxin (T4) and triiodothyronine (T3)). All patients with a diagnosis of thyroid cancer had been treated and were followed in the Department of Nuclear Medicine at the Johann Wolfgang Goethe-University Hospital and volunteered to participate in the study. After initial treatment with total thyroidectomy, we use a modification of the protocol described by Schlumberger (1998) in the follow-up of patients with thyroid cancer.

### Blood samples and RNA extraction

The interval between surgery and collecting of blood samples was > 3 months in all patients, all patients had received one cycle of radioiodine minimum and were taking L-T4. If there were thyroid residues detectable by radioscanning or the tumour was > pT2, an additional cycle of radioiodine was performed within 6 months. Approximately 9 ml venous blood was obtained from each patient in the morning prior to any diagnostic procedure in our outpatient clinic at a routine appointment. Blood samples were immediately mixed with EDTA to prevent coagulation and cooled to 4°C. Nucleated cells were isolated using a Percoll gradient (Pharmacia, Uppsala, Sweden) at a density of 1.09 g $ml^{-1}$ within 2 h after withdrawal of blood. Serum for thyroglobulin measurements was obtained using the supernatant after centrifugation and serum thyroglobulin was determined with a commercial immunometric assay (DYNOtest, Brahms Diagnostica, Berlin, Germany). A serum thyroglobulin level ≥ 5 ng $ml^{-1}$ (on L-T4 therapy) was considered elevated. The cell suspension was washed once in 0.9% sodium chloride and remaining erythrocytes were lysed. Total cellular RNA was extracted using RNeasy blood kit (Qiagen, Hilden, Germany) according to the suggestions of the manufacturer and RNA was resuspended in 50 µl RNase-free water. Optical transmission of the final RNA-preparation was determined three times at 260 nm using a Gene-Quant calculator (Pharmacia Biotech, Freiburg, Germany) and the concentration was calculated. The RNA suspension was directly used for RT-PCR or immediately stored in liquid nitrogen until use.

### Oligonucleotide primers and probes

Thyroglobulin primers were custom designed according to the human thyroglobulin cDNA sequence reported by Malthiery and Lissitzky (1987) (Gene Bank accession number U93033). All primers were synthesized and purified by MWG-Biotech (Ebersberg, Germany). These primers bind exon 9 and 10 respectively and span a genomic DNA length of 411 kb so that cDNA and contaminating genomic DNA amplification products can be distinguished by size (219 bp vs 630 bp). To assess the intactness of amplifiable RNA two intron-spanning glyceraldehyde-3-phosphate-dehydrogenase (GAPDH) primers that generate a 300 bp fragment (Gene Bank accession number M33197) were used. The specificity of the primer sets for thyroglobulin and glyceraldehyde-3-phosphate-dehydrogenase, respectively, was verified by basic local alignment search tool (BLAST) computer search (Altschul et al, 1990). The sequences of these PCR primers are listed in Table 1.

### Reverse transcriptase-polymerase chain reaction

Approximately 1.5 µg of total RNA was reverse transcribed in a final volume of 20 µl containing 4 µl 5 × buffer (250 mM Tris-HCl pH 7.5, 375 mM KCl, 15 mM $MgCl_2$, 50 mM DTT), 20 units recombinant RNase inhibitor (Promega, Madison, WI, USA), 0.5 mM dNTP, 2.5 µM oligo dT and 200 units RNase H minus M-MLV reverse transcriptase (Promega). The following PCR was performed in a total volume of 50 µl containing 3 µl of the RT-solution, 5 µl 10 × reaction buffer (200 mM Tris-HCl pH 8.4, 500 mM KCl), 0.5 µM forward and reverse primer, 0.2 µM of each dNTP, 2.5 units Taq-DNA polymerase and 1.5 mM $MgCl_2$ (Life Technology, Paisley, Scotland) using a tube-controlled DNA thermal cycler (AGS-Hybaid, Heidelberg, Germany). Samples were subjected to 3 min of denaturation at 94°C followed by 30 and 40 cycles respectively (normal and high RT-PCR sensitivity) of 45 s at 94°C, 30 s at 55°C and 1 min 30 s at 72°C, followed by additional 10 min at 72°C. In each experiment water was used as a negative control.

### Analysis of amplified thyroglobulin cDNA

10 µl PCR product was mixed with 2 µl 6 × loading dye (MBI Fermentas, Vilnius, Lithuania) and run for 60 min on a 1.5% agarose gel in TBE buffer (0.1 M Tris pH 8.4, 90 µM boric acid, 1 mM EDTA). The gel was stained with ethidium bromide and the bands corresponding to the amplified fragments were visualized on a UV table. Restriction analysis using SpeI (MBI Fermentas) was performed according to manufacturer's suggestions and sequencing was performed using a commercial sequencing service (SeqLab, Göttingen, Germany).

### Thyroglobulin RT-PCR assay sensitivity

RT-PCR assay sensitivity was determined by mixing total RNA isolated from whole blood of a negative control patient (negative with normal and high RT-PCR sensitivity) with total RNA isolated from normal human thyroid tissue in serial dilutions of 1:2, 1:5 and 1:10 steps, respectively. RT-PCR was performed and analysed

© 2000 Cancer Research Campaign as described above. Assuming that one thyrocyte contains approximately 10 pg total RNA, the lower detection limit of the described RT-PCR assay was calculated as thyroid cells/ml whole blood.

### Tissue specificity of TG-expression

Various human tissues, obtained from open surgery and/or necropsy were immediately frozen in liquid nitrogen and stored at –80°C until extracted. 100 mg of each frozen tissue sample was homogenized in 1 ml Tristar-reagent (AGS, Heidelberg, Germany) and total RNA was isolated according to suggestions of the manufacturer and the final RNA preparation was determined as described. After addition of 20 units RNase inhibitor (Promega) to prevent RNA degradation, the aliquots were stored in liquid nitrogen until use. RNA was extracted from the following tissues and organs: cerebrum, pituitary gland, palatine tonsil, thyroid, lung, heart, thymus, lymphatic node (located paratracheally), liver, gall bladder, spleen, pancreas, oesophagus, stomach, duodenum, jejunum, ileum, vermiform appendix, colon, kidney, suprarenal gland, urinary bladder, prostate, seminal vesicle, testes, tongue, muscle, ovary. In addition, two human thyroid carcinoma cell lines (B-CPAP and FTC 133) were analysed for TG mRNA expression. RT-PCR was performed and analysed as described above.

### Statistics

Statistical comparisons were done using Sigma-Stat statistical software (Jandel Scientific, Erkrath, Germany). Contingency tables were used to assess the association between RT-PCR positivity rates, research subject category and serum TG levels, respectively. Fisher exact test was used to compare the distributions in contingency tables that had five or less expected observations in one or more cells. Chi-square analysis was used to compare the distributions in contingency tables that had five or more observations expected in each cell. $P < 0.05$ was considered significant.

### RESULTS

### Detection of thyroglobulin transcripts by RT-PCR

After optimization of conditions for reverse transcription and polymerase chain reaction we were able to detect thyroglobulin transcripts as the expected 219 bp amplicon in human thyroid tissue by agarose gel electrophoresis. Restriction enzyme digestion with SpeI resulted in two restriction fragments of the calculated size and thus confirmed the restriction site in the amplified fragment. In addition, the PCR product was sequenced and its identity was consistent with human thyroglobulin cDNA sequence (Gene bank accession number U93033). PCR amplification using total RNA isolated from human thyroid tissue without prior reverse transcription was performed as described above. PCR without RT was unsuccessful in amplifying the products predicted from the cDNA sequence.

### Sensitivity of RT-PCR assay

RT-PCR assay sensitivity was determined using mixed aliquots of total RNA isolated from human thyroid tissue and lymphocytes of a negative control patient. Assuming that one thyrocyte contains 10 pg total RNA, we could detect the equivalent of 50–80 thyroid cells ml$^{-1}$ blood after 30 cycles of PCR and 10–20 thyroid cells ml$^{-1}$ blood after 40 cycles by agarose gel electrophoresis (Figure 1). Thus, there was a 4- to 5-fold increase in sensitivity after 40 cycles compared with 30 cycles PCR.

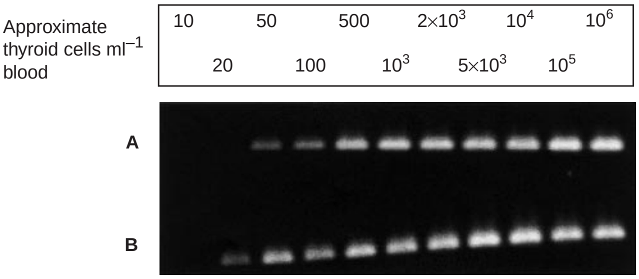

**Figure 1** Thyroglobulin RT-PCR sensitivity assay. Total RNA isolated from human thyroid tissue was mixed with total RNA from peripheral-blood mononuclear cells. (**A**) Sensitivity after 30 cycles of PCR amplification. By ethidium bromide staining, thyroglobulin mRNA equivalent to 50–80 cells ml$^{-1}$ blood was visualized. (**B**) Sensitivity after 40 cycles of PCR amplification. By ethidium bromide staining, thyroglobulin mRNA equivalent to 15–20 cells ml$^{-1}$ blood was visualized.

### Tissue specificity of TG-mRNA expression

To determine whether thyroglobulin mRNA expression is limited to and so specific for thyroid tissue, total RNA was extracted from various human tissues (listed in detail in Table 2) and RT-PCR was performed and analysed as described before using 30 and 40 cycles respectively. After 30 cycles of PCR, thyroglobulin mRNA expression was exclusively found in thyroid tissue. However, after 40 cycles of PCR, thyroglobulin mRNA expression was not only limited to thyroid tissue but also found in the thymus, suprarenal gland, hypophysis, lung, testis and vermiform appendix. Thus, thyroglobulin mRNA is a tissue-specific transcript after 30 cycles of PCR, but it is not after 40 cycles of PCR according to the described assay conditions.

### Evaluation of RT-PCR assay in patients and controls

We evaluated 150 patients with differentiated thyroid cancer (112 papillary, 38 follicular cancers), 137 of them without clinical signs of current metastasis, 13 with known metastasis, 29 patients with goitre, 19 patients with Hashimoto thyroiditis, 37 patients with Graves' disease, and 50 control patients without clinical signs or a history of thyroid disease. RT-PCR on all blood samples was performed using 30 and 40 cycles, respectively. Synthesis of cDNA was achieved in all cases, as determined by successful amplification of GAPDH cDNA in each sample.

After 30 cycles of PCR, 63/137 patients (45%) with treated thyroid cancer and no current metastasis were positive for thyroglobulin mRNA in the peripheral blood, 9/13 patients (69.2%) with metastasis, 7/29 patients (24%) with goitre, 5/19 patients (26%) with thyroiditis, 9/37 patients (23.7%) with Graves' disease, and 9/50 control patients (18%).

After 40 cycles of PCR, 111/137 patients (81%) with treated thyroid cancer and no current metastasis were positive for thyroglobulin mRNA in the peripheral blood, 11/13 patients (85%) with metastasis, 19/29 patients (66%) with goitre, 13/19 patients (68%) with thyroiditis, 29/37 patients (78%) with Graves' disease, and 41/50 control patients (82%). Results are summarized in Figure 2.

 *© 2000 Cancer Research Campaign*

**Table 2** Specificity of TG mRNA expression in different human tissues after 30 and 40 cycles of PCR, respectively

| Tissue | TG mRNA expression | |
|---|---|---|
| | 30 cycles | 40 cycles |
| Cerebrum | – | – |
| Hypophysis | – | + |
| Tongue | – | – |
| Palatine tonsil | – | – |
| Thyroid | + | ++ |
| Thymus | – | + |
| Lung | – | + |
| Heart | – | – |
| Lymph node | – | – |
| Liver | – | – |
| Gall bladder | – | – |
| Oesophagus | – | – |
| Stomach | – | – |
| Duodenum | – | – |
| Ileum | – | – |
| Appendix | – | + |
| Colon | – | – |
| Spleen | – | – |
| Pancreas | – | – |
| Kidney | – | – |
| Suprarenal gland | – | + |
| Urinary bladder | – | – |
| Seminal vesicle | – | – |
| Prostate | – | – |
| Testes | – | + |
| Ovary | – | – |
| Muscle | – | – |

Serum thyroglobulin values were available on all 150 patients with thyroid carcinoma and were determined in the serum supernatant of the cell preparation for total RNA. 137 patients of the above-mentioned had TG serum levels below 5 ng ml$^{-1}$ (mean 0.89 ng ml$^{-1}$ ± 0.075). All 13 patients with metastatic disease had TG serum levels above 5 ng ml$^{-1}$ (mean 173 ng ml$^{-1}$ ± 49.8). Patients with TG serum levels < 5 ng ml$^{-1}$ were RT-PCR positive in 45.9%, and patients with serum levels > 5 ng ml$^{-1}$ were RT-PCR positive in 69.2% of cases. Taken together, there was no significant correlation between serum thyroglobulin levels and detectable TG-mRNA ($P$ = 0.18, see Table 3).

### DISCUSSION

Using RT-PCR technique, we have developed an assay to detect thyroglobulin mRNA transcripts in the peripheral blood and have applied this assay to individuals with malignant and non-malignant thyroid disease, as well as to healthy controls. Molecular detection of tissue-specific gene expression in peripheral blood is a new diagnostic approach and has been described as a tumour marker in different solid tumours, such as prostate cancer (Moreno et al, 1992), neuroblastoma (Mattano et al, 1992), and breast cancer (Datta et al, 1994). Especially in prostate cancer, detection of circulating tumour cells could be shown to precede the detection of secondary disease by serum markers determined by immunoassays (Ghossein et al, 1995). To date, only three studies have been published, which have explored blood-borne thyroglobulin mRNA as a potential tumour marker in thyroid cancer (Ditkoff et al, 1996; Tallini et al, 1998; Ringel et al, 1998).

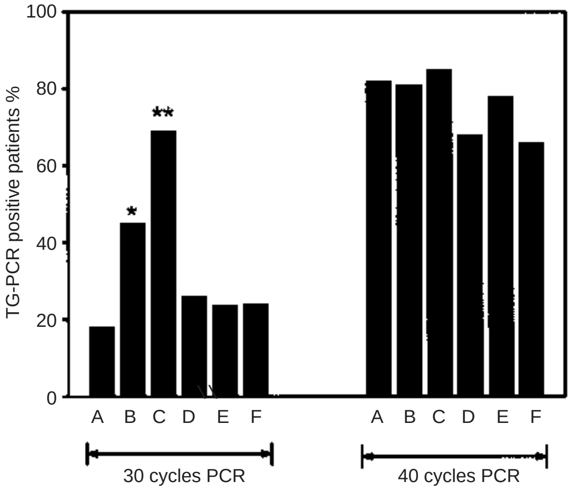

**Figure 2** TG mRNA positivity rates in research subjects categories. (**A**) controls, (**B**) patients with a history of thyroid cancer and no known metastasis, (**C**) patients with metastasis, (**D**) patients with thyroiditis, (**E**) patients with Graves' disease, (**F**) patients with goitre. All experiments were performed using 30 cycles of PCR amplification and 40 cycles of PCR amplification, respectively (*$P$ < 0.005 vs control, Chi-square test; ** $P$ < 0.005 vs control, Fisher exact test)

**Table 3** Correlation between TG mRNA detection and serum TG values in patients with and without metastasis (taken together) after 30 cycles of PCR. Category with a serum thyroglobulin > 5 ng ml$^{-1}$ includes all 13 patients with known metastasis. There was no significant correlation between serum thyroglobulin levels and detectable TG-mRNA ($P$ = 0.18)

| | | RT-PCR positive cases | |
|---|---|---|---|
| | Cases ($n$) | $n$ | % |
| Serum thyroglobulin > 5 ng ml$^{-1}$ | 13 | 9 | 69.2 |
| Serum thyroglobulin < 5 ng ml$^{-1}$ | 137 | 63 | 45.9 |

Using 'normal' RT-PCR sensitivity, PCR results were significantly more often positive in patients with thyroid cancer and metastasis (69%) compared with controls (18%). Interestingly, in patients with a history of thyroid cancer but no known current metastasis, PCR results were also significantly more often positive (46%) compared with controls. To date we cannot determine whether TG mRNA in these patients is falsely positive or these patients are not cured and will develop clinically-evident secondary disease. Therefore, a prospective follow-up study of the patients with a positive PCR result of TG mRNA expression in peripheral blood is now performed and this might add further information to clarify this important issue.

In contrast, frequency of RT-PCR positivity in control patients did not significantly differ from that in patients with goitre (24%), Graves' disease (24%) and patients with Hashimoto thyroiditis (26%). Interestingly, although they metastasize in a different manner – haematogen vs lymphogen (Simpson et al, 1987) – there was no detectable difference in PCR results between patients with follicular and papillary thyroid cancer, respectively. There was also no correlation between the status of lymph node involvement at date of surgery and PCR results. Multiple peripheral blood samples could be analysed for TG mRNA in two of the patients with known metastasis. Although TG serum levels were highly increased in both patients, even one negative RT-PCR result was obtained in both patients. The lack of detectable RT-PCR transcripts is difficult to explain, but on the other hand occurrence of negative peripheral blood samples in patients with metastatic disease has been noted in other tumour models, and intermittent release of tumour cells in the bloodstream may be one possible explanation (Ghossein and Rosai, 1996).

Although we observed a significant correlation between PCR positivity and the diagnosis of metastasized thyroid cancer with the described 'normal' assay sensitivity, results completely changed when applying 40 cycles of PCR instead of 30. Patients in all groups were positive for thyroglobulin mRNA in the peripheral blood in 66–85% without any significant difference or correlation between the single groups.

Until now, the clinical value of this RT-PCR approach could not definitively be determined and data from recent studies are partly inconsistent and contradictory (Haber, 1998). The application of this technique to detect recurrent thyroid cancer was first reported by Ditkoff et al (1996), who could demonstrate the presence of thyroglobulin mRNA transcripts in the peripheral blood in 9/9 patients with known metastatic thyroid cancer, 5/22 patients without current metastasis but a history of treated metastasis, 2/56 patients without current and no history of metastasis and 0/7 control volunteers.

Tallini et al (1998) reported that thyroglobulin mRNA could be detected in blood by RT-PCR in 4/20 patients with benign nodules, in 15/24 patients with treated thyroid cancer and 0/11 controls. However, they could detect TG mRNA in all of these controls as well as in 10 human cell lines when PCR amplification was extended from 30 to 40 cycles.

Finally Ringel et al (1998) recently reported an RT-PCR assay to detect blood-borne thyroglobulin mRNA. Of 14 patients with known metastasis based on radioscanning, all had detectable thyroglobulin mRNA in blood compared with 7/35 patients with negative radioiodine scans. Among 19 patients with residual radioiodine uptake in the thyroid bed, thyroglobulin mRNA was detectable in the blood of 12. Their assay was also positive in all 10 control subjects and the authors were able to identify these cells as putative circulating thyroid cells. Assay sensitivity was determined to detect 10 cells $ml^{-1}$ blood.

A variety of technical factors may contribute to the effect of different results obtained in the cited literature and our study. For example, different approaches were used to isolate total RNA from peripheral blood. Ditkoff et al (1996) used 'RNA-STAT 60' to extract total RNA, Tallini and coworkers used a Ficoll-gradient and Ringel et al extracted total RNA from whole blood with TRIzol™ reagent. Because of the best results in our hands, we used a Percoll-gradient to isolate nucleated cells from peripheral blood, followed by RNA extraction using a commercial extraction kit, but we were also able to detect thyroglobulin mRNA when extracting total RNA with TRIzol™ and – unlike Ringel et al – even when using a Ficoll-gradient.

To determine assay sensitivity, we used a technique comparable with the one reported by Ringel et al (1998). Initial experiments to measure assay sensitivity by mixing thyroid cells from human thyroid carcinoma cell lines (B-CPAP and FTC 133) with lymphocytes failed because of the very low level of TG mRNA expression in these cell lines. This phenomenon of decreased and possibly undetectable TG mRNA expression in differentiated thyroid carcinoma has already been described by other investigators (Hoang-Vu et al, 1992).

Ditkoff et al (1996) also used a human thyroid carcinoma cell line to determine assay sensitivity, but using unstimulated cells, only 200 cells $ml^{-1}$ blood were detectable compared with 20 cells $ml^{-1}$ blood when cells were stimulated with TSH. In view of these results and obvious technical limitation, assay sensitivities may reflect more approximate estimations than exact values and thus may be difficult to compare between the single studies.

Nevertheless, we could clearly show a dramatic effect of different assay sensitivities on the percentage of RT-PCR positive patients. After 30 PCR amplification cycles we achieved an assay sensitivity comparable to that reported by Tallini et al (1998) and in addition obtained similar results concerning TG mRNA positivity in patients. After 40 PCR amplification cycles we achieved an assay sensitivity comparable to that reported by Ringel et al (1998), but in contrast obtained a very high percentage of RT-PCR positive patients in all groups and thus completely different results without any significant differences between the groups or correlation with status of disease.

The reason for this high percentage of RT-PCR positive patients is unclear. Ringel et al were able to detect TSH-receptor and TG-positive cells by magnetic cell sorting in two studied control patients but not in one athyreotic patient and identified these cells as thyrocytes. In contrast, Tallini et al were able to detect TG mRNA transcripts in a variety of human cell lines and concluded that non-specific low level expression of a variety of genes in different cell types, or illegitimate transcription, may also occur in peripheral-blood leukocytes.

We favour the latter hypothesis, because after 40 cycles of PCR amplification, there was no difference in the percentage of positive PCR results irrespective of the thyroid status of the patients. In addition, we could also detect TG mRNA transcripts in the peripheral blood of two patients, who underwent total thyroidectomy due to medullary thyroid cancer, after 40 cycles but not after 30 cycles of PCR amplification. Therefore, detection of TG mRNA in peripheral blood at high assay sensitivities might reflect detection of illegitimate transcription in cell types of non-thyroid origin – e.g. lymphocytes – as it has been described earlier (Chelly et al, 1989).

In another set of experiments we tested whether TG mRNA expression is limited to and so specific for thyroid tissue. Tissue-specific expression of TG mRNA has not been determined in the earlier studies of Ditkoff et al (1996), Tallini et al (1998), and Ringel et al (1998). Although TG mRNA was exclusively detectable in thyroid tissue after 30 cycles of PCR amplification, it was also found to be expressed in the thymus, suprarenal gland, duodenum, lung, testis and appendix after 40 cycles of PCR amplification. In part, this phenomenon has already been described by other investigators (Aust et al, 1998). As a consequence, we have to assume that TG mRNA expression determined by RT-PCR is specific for thyroid cells using a normal assay sensitivity, but it is not specific using a high assay sensitivity.

In summary, with a normal RT-PCR sensitivity TG mRNA transcripts are specific for thyroid tissue and detectable in the peripheral blood of controls and patients with thyroid disease, which correlates with a diagnosis of cancer. However, with a high RT-PCR sensitivity, TG mRNA expression was found not to be specific for thyroid tissue and in addition was not correlated with a diagnosis of thyroid cancer. Furthermore, in patients with clinically known metastases there was no additional information of the PCR result of our assay compared with serum TG levels, which is part of the current 'gold standard' to detect disease recurrence (Herle and Uller, 1975; Gerfo et al, 1979).

 *© 2000 Cancer Research Campaign*

Taken together, the detection of circulating thyroid cancer micro-metastases in peripheral blood might be a new tool in clinical research. The high sensitivity of these methods, however, is combined with a possible decrease in specificity. Therefore, precaution is necessary to exclude artificial results. A standardization of cDNA preparation, gene-specific primer pairs, optimal RT-PCR conditions and the determination of possible threshold caused by cells of non-thyroid origin is mandatory to obtain reliable and comparable results. In view of our data obtained for sensitivity and specificity, we believe that a clinically useful assay will require further assay refinement, e.g. quantitative assay to permit the distinction of weakly and more strongly-positive RT-PCR results. A number of questions remain to be answered and further studies are necessary to determine how many of the patients without known metastasis and normal TG serum levels but a positive PCR result have or will develop secondary disease.

### REFERENCES

Altschul SF, Gish W, Miller W, Myers EW and Lipman DJ (1990) Basic local alignment search tool. *J Mol Biol* **215**: 403–410

Aust G, Crisp M, Bosenberg E, Ludgate M, Weetman AP and Paschke R (1998) Transcription of thyroid autoantigens in non-expressing tissues. *Exp Clin Endocrinol Diabetes* **106**: 319–323

Bellantone R, Lombardi CP, Boscherini M, Ferrante A, Raffaelli M, Rubino F, Bossola M and Crucitti F (1998) Prognostic factors in differentiated thyroid carcinoma: a multivariate analysis of 234 consecutive patients. *J Surg Oncol* **68**: 237–241

Chelly J, Concordet JP, Kaplan JC and Kahn A (1989) Illegitimate transcription: transcription of any gene in any cell type. *Proc Natl Acad Sci USA* **86**: 2617–2621

Datta YH, Adams PT, Drobyski WR, Ethier SP, Terry VH and Roth MS (1994) Sensitive detection of occult breast cancer by the reverse-transcriptase polymerase chain reaction. *J Clin Oncol* **12**: 475–482

Ditkoff BA, Marvin MR, Yemul S, Shi YJ, Chabot J, Feind C and Gerfo PL (1996) Detection of circulating thyroid cells in peripheral blood. *Surgery* **120**: 959–965

Gerfo PL, Colacchio T, Colacchio D and Feind C (1979) Thyroglobulin in benign and malignant thyroid disease. *JAMA* **241**: 923–924

Ghossein RA and Rosai J (1996) Polymerase chain reaction in the detection of micrometastases and circulating tumor cells. *Cancer* **78**: 10–16

Ghossein RA, Scher HI, Gerald WL, Kelly WK, Curley T, Amsterdam A, Zhang ZF and Rosai J (1995) Detection of circulating tumor cells in patients with localized and metastatic prostatic carcinoma: clinical implication. *J Clin Oncol* **13**: 1195–1200

Haber RS (1998) Editorial: The diagnosis of recurrent thyroid cancer – a new approach. *J Clin Endocrinol Metab* **83**: 4189–4190

Herle AJ and Uller RP (1975) Elevated serum thyroglobulin. A marker of metastases in differentiated thyroid carcinomas. *J Clin Invest* **56**: 272–277

Hoang-Vu C, Dralle H, Scheumann G, Maenhaut C, Horn R, von zur Mühlen and Brabant G (1992) Gene expression of differentiation and dedifferentiation markers in normal and malignant human thyroid tissues. *Exp Clin Endocrinol Diabetes* **100**: 51–56

Johnson PWM, Burchill SA and Selby PJ (1995) The molecular detection of circulating tumor cells. *Br J Cancer* **72**: 268–276

Loh KC, Greenspan FS, Gee L, Miller TR and Yeo P (1997) Pathological tumor-node-metastasis (pTNM) staging of papillary and follicular thyroid carcinomas: a retrospective analysis of 700 patients. *J Clin Endocrinol Metab* **82**: 3553–3562

Malthiery Y and Lissitzky S (1987) Primary structure of human thyroglobulin deduced from the sequence of its 8448-base complementary DNA. *Eur J Biochem* **165**: 491–498

Mattano LA, Moss TJ and Emerson SG (1992) Sensitive detection of rare circulating neuroblastoma cells by the reverse transcriptase-polymerase chain reaction. *Cancer Res* **52**: 4701–4705

Moreno JG, Croce CM, Fischer R, Monne M, Vihko P, Mulholland SG and Gomella LG (1992) Detection of hematogenous micrometastasis in patients with prostate cancer. *Cancer Res* **52**: 6110–6112

Noguchi M, Katev N and Miwa K (1998) Therapeutic strategies and long-term results in differentiated thyroid cancer. *J Surg Oncol* **67**: 52–59

Parker SL, Tong T, Bolden S and Wingo PA (1997) Cancer statistics, 1997. *Cancer J Clin* **47**: 5–27

Ringel MD, Ladenson PW and Levine MA (1998) Molecular diagnosis of residual and recurrent thyroid cancer by amplification of thyroglobulin messenger ribonucleic acid in peripheral blood. *J Clin Endocrinol Metab* **83**: 4435–4442

Sanders LE and Cady B (1998) Differentiated thyroid cancer: reexamination of risk groups and outcome of treatment. *Arch Surg* **133**: 419–425

Schlumberger M, Tubiana M, De Vathaire F, Hill C, Gardet P, Travagli JP, Fragu P, Lumbroso J, Caillou B and Parmentier C (1986) Long-term results of treatment of 283 patients with lung and bone metastases from differentiated thyroid carcinoma. *J Clin Endocrinol Metab* **63**: 960–967

Schlumberger MJ (1998) Papillary and follicular thyroid carcinoma. *N Engl J Med* **338**: 297–306

Simpson WJ, McKinney SE, Carruthers JS, Gospodarowicz MK, Sutcliffe SB and Panzarella T (1987) Papillary and follicular thyroid cancer. *Am J Med* **83**: 479–488

Tallini G, Ghossein RA, Emanuel J, Gill J, Kinder B, Dimich AB, Costa J, Robbins R, Burrow GN and Rosai J (1998) Detection of thyroglobulin, thyroid peroxidase, and RET/PTC1 mRNA transcripts in the peripheral blood of patients with thyroid disease. *J Clin Oncol* **16**: 1158–1166

*© 2000 Cancer Research Campaign*
